# BrAPI—an application programming interface for plant breeding applications

**DOI:** 10.1093/bioinformatics/btz190

**Published:** 2019-03-23

**Authors:** Peter Selby, Rafael Abbeloos, Jan Erik Backlund, Martin Basterrechea Salido, Guillaume Bauchet, Omar E Benites-Alfaro, Clay Birkett, Viana C Calaminos, Pierre Carceller, Guillaume Cornut, Bruno Vasques Costa, Jeremy D Edwards, Richard Finkers, Star Yanxin Gao, Mehmood Ghaffar, Philip Glaser, Valentin Guignon, Puthick Hok, Andrzej Kilian, Patrick König, Jack Elendil B Lagare, Matthias Lange, Marie-Angélique Laporte, Pierre Larmande, David S LeBauer, David A Lyon, David S Marshall, Dave Matthews, Iain Milne, Naymesh Mistry, Nicolas Morales, Lukas A Mueller, Pascal Neveu, Evangelia Papoutsoglou, Brian Pearce, Ivan Perez-Masias, Cyril Pommier, Ricardo H Ramírez-González, Abhishek Rathore, Angel Manica Raquel, Sebastian Raubach, Trevor Rife, Kelly Robbins, Mathieu Rouard, Chaitanya Sarma, Uwe Scholz, Guilhem Sempéré, Paul D Shaw, Reinhard Simon, Nahuel Soldevilla, Gordon Stephen, Qi Sun, Clarysabel Tovar, Grzegorz Uszynski, Maikel Verouden, The BrAPI consortium

**Affiliations:** 1 Plant Breeding and Genetics Section, School of Integrative Plant Science, Cornell University, Ithaca, New York, USA; 2 VIB, Ghent, Belgium; 3 Integrated Breeding Program (IBP), CIMMYT, Texcoco, Mexico; 4 Leibniz Institute of Plant Genetics and Crop Plant Research (IPK), Gatersleben, Germany; 5 Boyce Thompson Institute, Ithaca, NY, USA; 6 International Potato Center (CIP), Lima, Peru; 7 International Food Policy Research Institute (IFPRI), Washington DC, USA; 8 USDA ARS, Ithaca, NY, USA; 9 International Rice Research Institute (IRRI), Los Baños, Laguna, The Philippines; 10 AGAP, Univ Montpellier, CIRAD, INRA, Montpellier SupAgro, Montpellier, France; 11 URGI, INRA, Université Paris-Saclay, Versailles, France; 12 Instituto de Biologia Experimental e Technologica (iBET), Oeiras, Portugal; 13 USDA ARS, Stuttgart, AR, USA; 14 Department of Plant Breeding, Wageningen University & Research, Wageningen, The Netherlands; 15 Institute of Biotechnology, Cornell University, Ithaca, New York, USA; 16 Bioversity International, Montpellier, France; 17 Diversity Arrays Technology, Bruce, Australia; 18 DIADE, IRD, University of Montpellier, Montpellier, France; 19 College of Agricultural and Life Sciences, The University of Arizona, Tucson, AZ, USA; 20 Information & Computational Sciences, The James Hutton Institute, Dundee, UK; 21 SRUC, Edinburgh, UK; 22 LeafNode Technology, Auckland, New Zealand; 23 MISTEA, INRA, Montpellier SupAgro, Universite de Montpellier, Montpellier, France; 24 John Innes Centre, Norwich Research Park, Norwich, UK; 25 International Crops Research Institute for the Semi-Arid Tropics (ICRISAT), Hyderabad, India; 26 Department of Plant Pathology, Kansas State University, Manhattan, KS, USA; 27 INTERTRYP, Univ Montpellier, CIRAD, IRD, Montpellier, France; 28 Patranca E.I.R.L., Lima, Peru; 29 LeafNode Technology, Buenos Aires, Argentina; 30 Wageningen University & Research, Biometris, Wageningen PB, The Netherlands

## Abstract

**Motivation:**

Modern genomic breeding methods rely heavily on very large amounts of phenotyping and genotyping data, presenting new challenges in effective data management and integration. Recently, the size and complexity of datasets have increased significantly, with the result that data are often stored on multiple systems. As analyses of interest increasingly require aggregation of datasets from diverse sources, data exchange between disparate systems becomes a challenge.

**Results:**

To facilitate interoperability among breeding applications, we present the public plant Breeding Application Programming Interface (BrAPI). BrAPI is a standardized web service API specification. The development of BrAPI is a collaborative, community-based initiative involving a growing global community of over a hundred participants representing several dozen institutions and companies. Development of such a standard is recognized as critical to a number of important large breeding system initiatives as a foundational technology. The focus of the first version of the API is on providing services for connecting systems and retrieving basic breeding data including germplasm, study, observation, and marker data. A number of BrAPI-enabled applications, termed BrAPPs, have been written, that take advantage of the emerging support of BrAPI by many databases.

**Availability and implementation:**

More information on BrAPI, including links to the specification, test suites, BrAPPs, and sample implementations is available at https://brapi.org/. The BrAPI specification and the developer tools are provided as free and open source.

## 1 Introduction

Plant breeding is widely recognized as crucial to feeding a rapidly growing population, especially in developing countries (Flavell, 2017), (http://www.fao.org/fileadmin/templates/wsfs/docs/expert_paper/How_to_Feed_the_World_in_2050.pdf). To meet this demand, it is necessary to breed new varieties that maintain high productivity with reduced inputs and are adapted to new eco-agricultural environments resulting from climate change. Plant breeding is a complex undertaking that necessarily integrates many interrelated disciplines, each with their own conventions for data structure and storage, and increasingly large, multi-faceted datasets.

To address the challenges in the size and complexity of breeding data, a number of database systems have been designed over the years to solve specific problems. Although the power and insights that can be gleaned from large datasets increase with a greater volume and diversity of data sources, these separate systems make data integration difficult. Breeders need seamless access to all relevant data, but each system tends to keep its data siloed with *ad hoc* formats that hinder the ability to exchange, compare and combine data across research teams.

To meet these requirements, numerous groups have been working together to create an Application Programming Interface (API) for breeding data (Ghouila *et al.*, 2018). An API specification describes the functions and services available in an application which can be accessed in an automated way by a computer program. It describes what services are available, what inputs are allowed, what the structure of the output data will be, and the protocol used to pass data to a service, often on the web. In recent years, web services have become the major paradigm for information exchange on the web, and web service standards have also been defined and implemented successfully by the bioinformatics community. Examples of such systems include the Distributed Annotation System (DAS) (Dowell *et al.*, 2001), BioMOBY (Wilkinson and Links, 2002), and the EMBRACE (Pettifer *et al.*, 2010) Web Service collection.

Most of the modern web service infrastructure follows the REST standards (Fielding and Taylor, 2002). REST stands for ‘Representational State Transfer’ and defines a stateless client/server communication architecture, built on the HyperText Transfer Protocol (HTTP) (https://tools.ietf.org/html/rfc7231). In a RESTful API, HTTP is the communication protocol and the available services are defined as Unified Resource Locators (URLs). Typically, the inputs are defined by constructing a URL with query parameters defined by the API (or HTTP request body objects for more complex inputs), the output data are usually returned in a defined structure. For the output, historically, XML was used, but newer APIs typically prefer the Javascript Object Notation (JSON) format.

Data exchange requires solutions on many levels, including the semantic level and the syntactic level (Doan *et al.*, 2004). For breeding data, standardization of the semantic level has made significant progress over the last few years through the definition of ontologies for describing plant structure and development (Cooper *et al.*, 2018), and for describing traits in popular crops (Shrestha *et al.*, 2012). However, the breeding community still needs to standardize data at the syntax level. This can be achieved by defining a standardized Application Program Interface.

Here, we report on the design and implementation of a standard RESTful Breeding API (BrAPI), as a specification with a focus on common plant breeding data requirements. The interface was designed by members of the BrAPI consortium. A complete list of contributors is given in the consortium description and a continuously updated list can be found on the BrAPI website (https://brapi.org/).

## 2 Results

The Breeding API is a practical tool to help solve problems in accessing, exchanging, and integrating data across systems and applications. Given the multidisciplinary nature of plant breeding, there is a broad range in the particulars of the possible data operations that could be considered. Since a complete list of BrAPI related use cases would grow unmanageably large, we decided to focus on a small number of main use cases to design the primary API elements with a view towards reusability in other use cases.

### 2.1 Use cases

These are the main use cases we considered:

#### 2.1.1 Field phenotyping apps

Trials are often performed in fields that have limited internet connectivity, requiring special solutions for collecting phenotypic data. A popular approach is to collect data using handheld devices paired with custom mobile apps (Rife and Poland, 2014). Information about the field, the plot and accession identifiers needs to be loaded on the device before phenotypic data collection. After completion, collected data need to be uploaded to the database, when internet connectivity is available. Currently available solutions require custom files to be transferred, often involving significant user intervention. However, a simpler method would be to use an API to retrieve and store the data directly from the database.

#### 2.1.2 Sample tracking

For both phenotyping and genotyping applications, analyses may need to be run by service providers, such as analytical labs and genotyping centers, that use different tracking mechanisms. The sample tracking use case describes the hand-off of the sample information to the service provider, and the subsequent retrieval of the results. In practice, tracking samples can be complex because the identifiers from several different systems must be correlated.

#### 2.1.3 Genome visualization and analysis

Genome-based breeding requires extensive genotyping, which can be helpful to visualize in different ways to aid in breeding decisions. An example of such a tool is Flapjack (Milne *et al.*, 2010), which can display a number of genotypes and run analyses on the data. BrAPI standardizes the interfaces for such tools, hence they can be used with a much wider range of data sources and without the need for special adaptations for each source.

#### 2.1.4 FAIR data portals

One of the challenges of big data is identifying datasets of interest and ensuring their long term availability. This can be addressed by building federations of Findable, Accessible, Interoperable and Reusable (FAIR) data repositories (Wilkinson *et al.*, 2016). Interfaces such as BrAPI can help such efforts by standardizing access to the data repositories, thereby creating federations. Portals to the federated data can then be deployed to provide general or community specific data access. This increases the visibility of all datasets and therefore reduces the risk of losing isolated datasets over time. The portals should implement simple searches on standard metadata, such as MCPD or MIAPPE (Ćwiek-Kupczyńska *et al.*, 2016; Krajewski *et al.*, 2015; Milne *et al.*, 2010).

#### 2.1.5 Data integration and exchange

In this use case, two databases exist with overlapping data as well as specific data in each database. Database A would like to access data in database B. For example, database A may contain information about accessions, such as phenotypic and trial metadata, while database B contains genotypic information. Using a BrAPI call, database A can extract the genotyping data from database B and use that data in breeding decision support. 

### 2.2 API definition

The BrAPI definition is kept in the ‘API’ repository of the ‘plantbreeding’ organization on GitHub (https://github.com/plantbreeding/API), with all changes to the definition managed using GitHub’s ‘issues’, ‘projects’ and ‘pull requests’ facilities.

#### 2.2.1 API organization

BrAPI calls are organized into categories that reflect the major domains needed for exchanging data between plant breeding information management systems and client applications. Some example categories include Studies, Germplasm, Traits, Trials, MarkerProfiles and Authentication. (A full list of the categories is presented in Table 1.)

**Table 1.** Categories of BrAPI calls

Category	Comments	# of calls
Calls	Meta information about which BrAPI calls are available on a server implementation.	1
Crops	Provides the common names for the crops available on a server implementation.	1
Germplasm	Provides search capabilities and details for germplasm data. Includes MCPD, pedigree and breeding method data.	8
Germplasm Attributes	Germplasm Attributes are simply inherited characterization descriptors that are inherent in the germplasm line but not environment-dependent.	3
Markers	Provides search capabilities and details for genetic marker metadata.	3
Marker Profiles	Provides search capabilities and details for genomic data. Includes allele matrices.	5
Programs	Provides search capabilities and details for breeding programs. A program may contain multiple trials.	2
Trials	Provides search capabilities and details for breeding trials. A trial may contain multiple studies. Used also for any large phenotyping dataset like multilocal phenotyping networks.	2
Studies	Provides search capabilities and details for genotyping and phenotyping studies and support for observation data gathering. Includes germplasm, observation, plot layout, and season details related to a particular study.	17
Phenotypes	Provides search capabilities for phenotyping observation data across studies, trials, and programs	5
Traits	Provides details for trait ontology data which are available for observation variables.	2
Observation Variables	An Observation Variable is combination of a trait, a method and a scale. Phenotyping data are collected for observation variables. Fully aligned to the Crop Ontology.	5
Genome Maps	Provides summaries and details for stored genome maps.	4
Location	Provides details of geographical locations of studies.	2
Samples	Provides support for storing and retrieving plant sample metadata	4
Vendor Samples	Provides support for sending sample metadata to an external vendor for processing (ie gene sequencing)	5

*Note*: In each category, there are one or more calls that provide services to support the corresponding domain of plant breeding data management.

#### 2.2.2 URL structure

All BrAPI calls follow a common URL structure. The URL starts with a domain name (and optional base path of the implementation server) followed by ‘/brapi/’ and the major version number. Next, the call name appears with optional object ids and other parameters. Most calls use the HTTP request method ‘GET’, but some require ‘POST’ and ‘PUT’, as specified in the documentation. For security, the use of SSL (HTTPS) is highly recommended for all BrAPI endpoints.

Examples:

https://example.com/brapi/v1/locations

https://example.com/brapi/v1/trials? programDbId=abc123

https://example.com/maize-db-01/brapi/v1/studies-search

#### 2.2.3 Return object structure

**Fig. 1.**
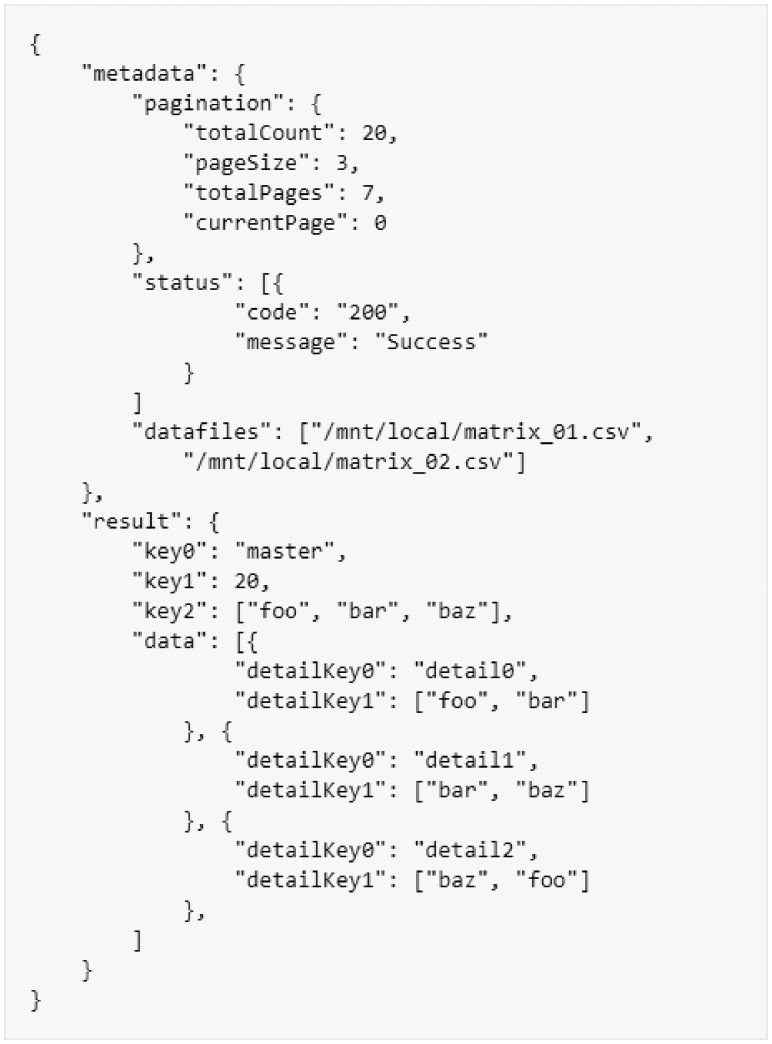
An example BrAPI response object. This object shows a generic response with ‘metadata’, a ‘master’ result record and a set of ‘data’ records

We have defined a standard JSON formatted response structure that is common across all calls. The standard response consists of a JSON object with a ‘metadata’ key and a ‘result’ key. The ‘metadata’ key provides the pagination information, an array of status information, and an array of data files. If the response data contain an array of entities which could possibly grow large, the ‘pagination’ object will be populated with the keys ‘pageSize’, ‘currentPage’, ‘totalCount’, ‘totalPages’ containing the appropriate values. If the response is a single entity that does not require pagination, then the ‘pagination’ object still must be returned, but all data elements within it should be set to zero. All pages are zero indexed, so the first page will always be page zero. The ‘status’ array contains a list of objects with the keys ‘code’ and ‘message’. These status objects should be used to provide additional status or log information about the call. If the call was completed successfully and there are no status objects reported, an empty array should be returned. The ‘datafiles’ array contains a list of URLs to any extra data files generated by the call. For example, this could be images related to the data returned, or large data extract files which contain more data than that returned in the response payload, see Figure 1 for an example.

The data payload ‘result’ contains the specific model object for the given call response. There are three basic patterns that response objects follow. The ‘master’ pattern is used for returning all the data associated with a single entity. The ‘details’ pattern is used to return an array of entities. In the ‘details’ pattern, the ‘result’ object always contains a single array called ‘data’ and no other fields. The ‘master/details’ pattern is a combination of the ‘master’ and ‘details’ patterns. It is used to represent a parent object which has an array of child entities. The ‘result’ object contains some data associated with the parent as well as the ‘data’ array with all the child entities. Whenever the ‘data’ array is present, the response is assumed to be paginated. This means the size of the ‘data’ array is always limited by the ‘pagination’ object in the ‘metadata’.

In most cases, all the data will be contained within the JSON response. For large ‘data’ arrays, several requests might need to be made to retrieve every page of the array. In the event that the size of the data package exceeds what could reasonably be handled using the HTTPS protocol and the client, the service provider can place the data in a file and provide a link in the ‘datafiles’ array, to be downloaded later.

### 2.3 Authentication

A user or system may have to authenticate to a server to access protected data. BrAPI is a data communication specification, so the authentication scheme used to protect that data is considered outside the scope of the BrAPI specification. However, authentication and authorization are important topics to address whenever any kind of data is moved or presented. In order to facilitate communication of data between tools in a standardized way, the BrAPI community has developed a set of best practices using the OAuth2 architecture for implementing proper authentication with any BrAPI enabled tools and databases. In its most basic form, the OAuth2 architecture is a sessionless, token based architecture. OAuth2 allows users to sign in with user credentials they already have, and provides a token. This token can then be used to authenticate that user within different tools and databases. The token should be added as a header in every BrAPI request.

### 2.4 Versioning

All software projects need the ability to evolve to reflect changing requirements, to cover new use cases, and to incorporate user feedback. A well defined and rigorous versioning scheme is essential for BrAPI to ensure that client and server communication is well defined and the community can keep track of the changes. In BrAPI, there are major versions and minor versions. The major version is currently ‘v1’, which is reflected in the URL scheme. Minor versions are incremented about every three months, reflecting changes in the API that have been accepted by the BrAPI community and reviewed by the BrAPI coordinator. To help maintain consistency, all changes in minor versions are backward compatible with earlier minor versions within the same major version. The ‘calls’ call provides meta-information about each BrAPI call available on a given server. The response of the ‘calls’ call includes all the supported version numbers for each call, so external clients can easily check for compatibility with that server.

### 2.5 Community

For a communication standard like BrAPI to be successful, there must be people and organizations willing to contribute and use it. Early on in the development of BrAPI, we recognized the need to foster and develop a strong community of users. This community has grown rapidly over the past few years and it now has representatives from several dozen different organizations from around the world.

The development of BrAPI is a community effort. Work on the API is mainly organized around regular ‘hackathons’, where BrAPI contributors gather for a week of discussions and API design work. BrAPI community institutions take turns organizing and hosting the hackathons. This has proven very effective for collaborative development and capacity building (Ghouila *et al.*, 2018). Between the hackathons, the proposed APIs are implemented at the different sites, and problems encountered during implementation are fed back into the design at the following hackathon. An important role in the community is played by the BrAPI coordinator, who helps to organize the hackathons and workshops, reviews and coordinate proposals for new or updated calls, provides support for implementers, and maintains the documentation and the BrAPI website.

#### 2.5.1 Brapi.org

To serve the developer community, a website (https://brapi.org) was created as a nexus of all BrAPI related tools and information. It provides the official documentation for the API as well as information on meetings, hackathons, community news, testing tools, development libraries, BrAPPs, and a community forum.

### 2.6 Server implementations

BrAPI server implementations have been created for a number of popular breeding, genebank and plant genomics databases. A variety of languages and database systems have been used to develop BrAPI-compliant systems. Web frameworks’ languages include Drupal/Tripal (PHP), Catalyst (Perl), Java Spring (Java), NodeJS (JavaScript), Django (Python) whereas databases and data query systems include Postgres, MongoDB, Elasticsearch, HDF5, and MySQL. Many of these systems are open source, so their code may be adapted for other systems with similar implementation parameters. A list of current BrAPI server implementations is given in Table 2.

**Table 2.** Server implementations

Database name	URLs	Organization, Reference
Breeding Management System (BMS)	https://www.integratedbreeding.net	CGIAR https://cgiar.org
	Description: comprehensive breeding management system with trial design, data collection, and analyses.	
Cassavabase Musabase Yambase Sweetpotatobase Solanaceae Genomics Network	https://cassavabase.org https://musabase.org https://yambase.org https://sweetpotatobase.org https://solgenomics.net	Boyce Thompson Institute (BTI) https://btiscience.org
	Description: comprehensive breeding management system, including trial design management, phenotyping sample and data collection; with a focus on genomic breeding technologies such as Genomic Selection	
B4R	https://b4r.irri.org	International Rice Research Institute (IRRI), http://irri.org
	Description: comprehensive breeding management system tailored for rice and other grains	
Germinate	https://ics.hutton.ac.uk/get-germinate	The James Hutton Institute, http://hutton.ac.uk
	Description: breeding database and analysis tools	
GOBii	http://gobiiproject.org	Cornell University, https://cornell.edu BTI, https://btiscience.org
	Description: large scale and efficient genotyping storage system including data analysis workflows	
T3	https://triticeaetoolbox.org	USDA, https://usda.gov
	Description: comprehensive breeding management system designed for wheat	
Musa Germplasm Information System (MGIS)	https://www.crop-diversity.org/mgis	Bioversity International, https://bioversityinternational.org, (Ruas *et al.*, 2017)
	Description: information system on banana germplasm	
Gigwa	http://gigwa.southgreen.fr	CIRAD, IRD (South Green)
	Description: Gigwa (Sempéré *et al.*, 2016) is a web-application that aims at storing and exposing genotypic datasets and provides a web interface for filtering them in real time. It is able to interoperate with genome browsers and export results into several formats.	
EU-SOL Database	https://www.eu-sol.wur.nl	Wageningen University & Research, https://wur.nl
	Description: this site contains information about a collection composed of ∼7000 domesticated (S. lycopersicum) lines, along with representative wild species, collected with the scope of the european project EU-SOL. This germplasm was generously provided by different international genebanks and by donations from private collections. This Integrated Project is supported by the European Commission through the 6th framework program. Contract number: FOOD-CT-2006-016214	
GnpIS	https://urgi.versailles.inra.fr/gnpis	INRA, https://www.inra.fr
	Description: French national archive for plant phenotyping data. It provides any type of PGR and Phenotyping data. Used for instance by Perpheclim for climate change adaptation studies and as a data repository in the Elixir federation which is under construction. It contains almost a thousand Phenotyping trials over thousands of woody and annual plant varieties.	
KDDart	https://kddart.diversityarrays.com/brapi/v1/	DArT, http://www.kddart.org
	Description: genotype and phenotype database, linked to genotyping service	
Crop Ontology	http://www.cropontology.org/	Bioversity, https://bioversityinternational.org
	Description: database of available trait ontologies for diverse crops in the CGIAR system	
PIPPA	https://pippa.psb.ugent.be	VIB https://www.psb.ugent.be/
	Description: PSB Interface for Plant Phenotype Analysis	
PHIS	http://www.phis.inra.fr	INRA, https://www.inra.fr
	Description: ontology-driven Information System designed for Plant Phenomics. PHIS is designed to store, organize and manage highly heterogeneous and multi-spatial and temporal data from multiple sources (field, greenhouse).	
GBIS/I	https://fair-ipk.ipk-gatersleben.de/public/breedingapi.html	IPK-Gatersleben, https://www.ipk-gatersleben.de
	Description: among other, FAIR-IPK offers access to IPK genbank information system GBIS. This comprise passport data (information on the identity, history, geographical origin and botanical classification of the material) of the 150, 780 accessions in Gatersleben (as of 30 June 2016), including the Satellite Collections North in Gross Lüsewitz (potatoes) and Malchow/Poel (oil and fodder crops).	
TERRA REF	https://terraref.ncsa.illinois.edu/bety	https://terraref.org
	Description: an open access reference database for high throughput phenomics. Crops include sorghum and wheat.	

### 2.7 Client implementations

**Fig. 2.**
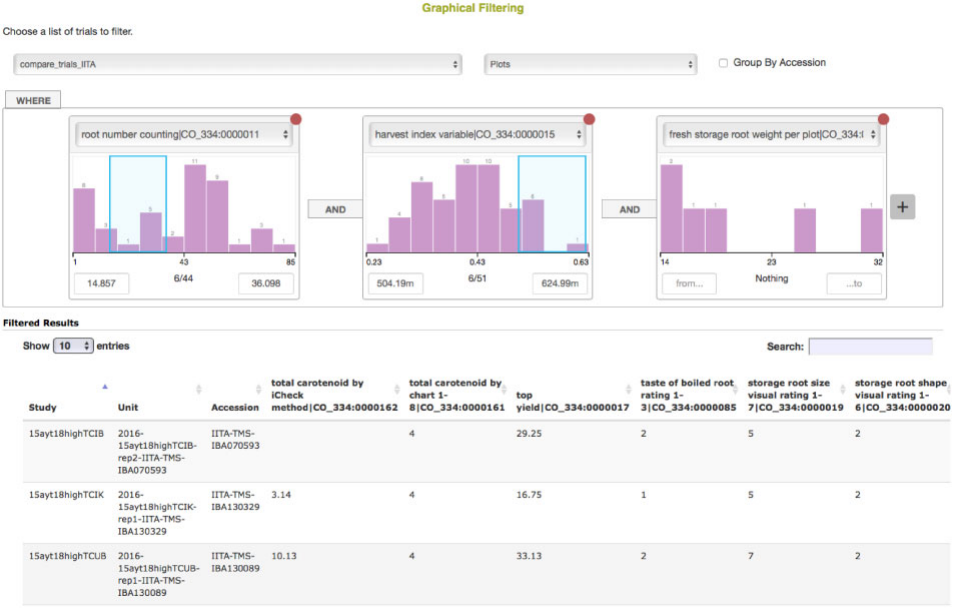
A screenshot of an example web application that retrieves information through BrAPI. Such applications are often referred to as ‘BrAPPs’. This application, called ‘Graphical Filtering’, allows to filter accessions by phenotypic data, by interactively selecting ranges of trait values for different traits in the dataset. Data from Cassavabase (https://cassavabase.org/) are shown, but BrAPPs seamlessly integrate with any BrAPI-enabled database

BrAPI client code libraries have been created in several languages, such as Java (https://github.com/imilne/jhi-brapi), the BrAPI R package (https://github.com/CIP-RIU/brapi), Brapi Drupal for PHP, and brapi.js for Javascript (https://github.com/solgenomics/BrAPI-js). A non-exhaustive list of current client applications is given in Table 3. It is possible for service providers to use BrAPI for the implementation of native website features. Some of these features have been implemented as reusable BrAPI compliant widgets, which we call BrAPI Apps or ‘BrAPPs’ for short. The available BrAPPs are listed on the BrAPI website (https://brapi.org/brapps.php). Figure 2 shows a screenshot of an example BrAPP which performs graphical filtering of phenotypic values.

**Table 3.** Client implementations

Program name	URL	Institution(s)
Flapjack	https://ics.hutton.ac.uk/flapjack	The James Hutton Institute, https://hutton.ac.uk
Highly Interactive Data Analysis Platform (HIDAP)	https://apps.cipotato.org/hidap_sbase/	International Potato Center (CIP)
brapi R package: Implementation of Breeding API in R	https://github.com/CIP-RIU/brapi	International Potato Center (CIP), Wageningen University & Research, Patranca
brapixR package	https://github.com/c5sire/brapix	Patranca
brapiui R package	https://github.com/c5sire/brapiui	Patranca
Pedigree Viewer	https://github.com/solgenomics	BTI
Graphical Phenotype Filtering	https://github.com/solgenomics	BTI
Trial Comparison	https://github.com/solgenomics	BTI
Comparative Map Viewer	http://maps.solgenomics.net/	BTI
ISMU	https://github.com/icrisatSbdm/ismu	ICRISAT
Gigwa	http://gigwa.southgreen.fr	CIRAD, IRD (South Green)
Beegmac	http://webtools.southgreen.fr/BrAPI/Beegmac/	CIRAD (South Green)
GnpIS	https://urgi.versailles.inra.fr/gnpis	INRA
Variable Ontology Widget	https://github.com/gnpis/trait-ontology-widget	INRA
Drupal BrAPI Implementation	https://www.drupal.org/project/brapi	Bioversity

### 2.8 Test suites and fixtures

Comprehensive testing is very important for any software project. Testing tools are available for both BrAPI server implementations and BrAPI enabled clients.

For testing BrAPI enabled clients, a BrAPI test server is available at the brapi.org site (https://test-server.brapi.org/brapi/v1). The BrAPI Test Server has a complete implementation of the BrAPI specification and returns a consistent sample set of data. This allows developers of clients to build tests which are appropriate for their tool, while calling a live BrAPI server implementation. The sample data reported by the test server are completely fabricated, and can be updated at any time upon request.

#### 2.8.1 BrAPI validator (Brava) test tool

**Fig. 3.**
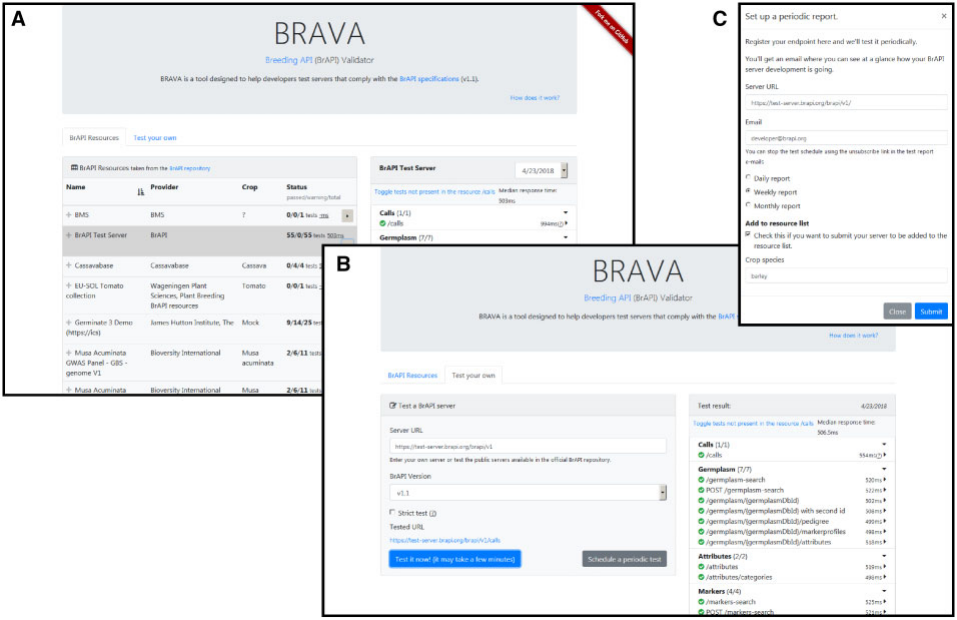
BRAVA portal. (**A**) List of publicly available endpoints and their compliance status according to BRAVA. An expanded report panel shows the individual test results for the selected resource. (**B**) ‘Test your own’ panel where the user can test a custom URL or (**C**) subscribe to get periodic reports

For testing server implementations, the BRAVA test client is available for testing compliance with the BrAPI specification (http://webapps.ipk-gatersleben.de/brapivalidator/). Available as a web frontend, BRAVA enables developers to check the compliance of their BrAPI endpoints against the specification and the referential integrity of input and output parameters of dependent endpoints. The frontend, as shown in Figure 3, enables testing of BrAPI server implementations. A user can also schedule tests and generate periodic reports of the overall status and details of BrAPI endpoint compliance. The compliance tests and results are grouped by and aggregated per REST resource. Using the BrAPI meta-endpoint ‘/calls’, BRAVA is able to detect the available endpoints on the server and will only test those endpoints.

A given endpoint might be tested multiple times with different inputs or HTTP methods. Each test checks the HTTP status code, content type, validity of response body, and response data types. Each test will also compare the response to the expected JSON schema which defines the structure of a JSON object and acceptable types. Some tests check the compatibility of response data to a corresponding parameter. For example, a test will call ‘/germplasm-search’ and will use the first ‘germplasmDbId’ from the response to make the call ‘/germplasm/{germplasmDbId}’. Some tests will compare a response value to a previously stored value. For example, an entity accessed by calling ‘/germplasm/1’ must have a ‘germplasmDbId’ of ‘1’.

When a test run is complete, the test suite result is sent to the web client and a report is generated. The report can be inspected in the client and has a tree-like structure to analyse results for individual calls. The scheduled and public resource test reports are stored for future assessment.

## 3 Discussion and Outlook

We have defined a first version of a plant breeding API that defines the key calls needed to exchange information about germplasm, phenotypes, experiments, studies, geographic locations, samples, and genetic markers. This opens the door to a rich set of possibilities for building client applications that can work with any BrAPI-compliant data provider.

Since 2015, a diverse group of data providers and client application programmers have been building BrAPI into their software. Client applications can rely on the standard interface to enable integration with any BrAPI data source. Building software using standard interfaces is an efficient and sustainable coding practice which enables the reuse of software components. As the public plant breeding software community is relatively small, this will be essential for creating a feature-rich breeding software ecosystem. A good example of the efficient reuse of components can be seen in the community developed BrAPPs, which are tools that make extensive use of BrAPI and can be widely shared and deployed on different BrAPI enabled systems. This framework is useful to commercial plant breeding software development efforts and we welcome more engagement with that community.

We are continuing our efforts and have initiated work on improved versions of the API. We recognize that the types of data relevant to plant breeding are expanding, and BrAPI will continue to evolve in response.

One aspect of the API that we would like to enhance is the ability to handle linked data (Xin *et al.*, 2018). For example, linking between datasets can rely on standard variables, vocabularies or ontologies, such as the Crop Ontology for Agricultural Data (Shrestha *et al.*, 2012). To fully enable this, current research and developments are based on adding semantic capabilities to BrAPI, especially through the JSON-LD standard, and some support will likely be included in the next major version of BrAPI. It is also important to improve the clarity and understandability of the BrAPI data for both human and machine. Future development will include documentation of the mapping between BrAPI and other common data specifications, such as MIAPPE and MCPD. This will provide a human friendly documentation of BrAPI formats and concepts. Furthermore, it will also provide reference concepts and schemas necessary to integrate BrAPI with other initiatives such as bioschemas.org.

Beyond breeding applications, BrAPI has also found a niche in gene bank applications, such as MGIS (Ruas *et al.*, 2017), through compatibility with the Multi-Crop Passport Data standard (MCPD). Although the initial intent was to enable interoperability between breeding management resources, BrAPI can also be used with other types of databases, such as plant genetic resources databases [i.e. MGIS (Ruas *et al.*, 2017)] and plant genome databases (i.e. SGN, MaizeGDB, etc.). BrAPI offers a way to link genetic resources distributed by gene banks with materials used in breeding programs. Improved integration between gene banks and plant breeding management databases, and genomic databases has the potential to greatly enhance the management and utilization of plant germplasm collections (Ruas *et al.*, 2017; Spindel and McCouch, 2016). Efficient and smart use of genetic diversity is a key for continued progress in plant breeding efforts to address the challenges of increased productivity and adaptation (Halewood *et al.*, 2018).

As the needs and technologies of our community continue to evolve, we expect BrAPI to grow to meet those needs.

### 3.1 Getting involved

We invite the reader to join our community and contribute to the future of BrAPI. To start, please visit https://brapi.org/ to learn more, contact the BrAPI coordinator at brapicoordinatorselby@gmail.com to join the mailing list, Slack channel, and community forum.

## References

[btz190-B1] Cooper L. et al (2018) The planteome database: an integrated resource for reference ontologies, plant genomics and phenomics. Nucleic Acids Res., 46, D1168–D1180. 29186578 10.1093/nar/gkx1152 PMC5753347

[btz190-B2] Ćwiek-Kupczyńska H.T. et al (2016) Measures for interoperability of phenotypic data: minimum information requirements and formatting. Plant Methods, 12, 44. 27843484 10.1186/s13007-016-0144-4 PMC5103589

[btz190-B3] Doan A. et al (2004) Introduction to the special issue on semantic integration. ACM SIGMOD Record, 33, 11.

[btz190-B4] Dowell R.D. et al (2001) The distributed annotation system. BMC Bioinformatics, 2, 7. 11667947 10.1186/1471-2105-2-7 PMC58584

[btz190-B5] Fielding R.T., Taylor R.N. (2002) Principled design of the modern web architecture. ACM Trans. Internet Technol., 2, 115–150.

[btz190-B6] Flavell R.B. (2017) Innovations continuously enhance crop breeding and demand new strategic planning. Glob. Food Sec., 12, 15–21.

[btz190-B7] Ghouila A. et al (2018) Hackathons as a means of accelerating scientific discoveries and knowledge transfer. Genome Res., 28, 759–765. 29650552 10.1101/gr.228460.117 PMC5932615

[btz190-B8] Halewood M. et al (2018) Plant genetic resources for food and agriculture: opportunities and challenges emerging from the science and information technology revolution. New Phytol., 217, 1407–1419. 29359808 10.1111/nph.14993

[btz190-B9] Krajewski P. et al (2015) Towards recommendations for metadata and data handling in plant phenotyping. J. Exp. Botany, 66, 5417–5427. 26044092 10.1093/jxb/erv271

[btz190-B10] Milne I. et al (2010) Flapjack–graphical genotype visualization. Bioinformatics, 26, 3133–3134. 20956241 10.1093/bioinformatics/btq580 PMC2995120

[btz190-B11] Pettifer S. et al (2010) The EMBRACE web service collection. Nucleic Acids Res., 38, W683–W688. 20462862 10.1093/nar/gkq297 PMC2896104

[btz190-B12] Rife T.W., Poland J.A. (2014) Field book: an open-source application for field data collection on android. Crop Sci., 54, 1624.

[btz190-B13] Ruas M. et al (2017) MGIS: managing banana (Musa Spp.) genetic resources information and high-throughput genotyping data. Database (Oxford), 2017, 29220435. 10.1093/database/bax046 PMC5502358 29220435

[btz190-B14] Sempéré G. et al (2016) Gigwa—genotype investigator for genome-wide analyses. Gigascience, 5, 25. 27267926 10.1186/s13742-016-0131-8 PMC4897896

[btz190-B15] Shrestha R. et al (2012) Bridging the phenotypic and genetic data useful for integrated breeding through a data annotation using the crop ontology developed by the crop communities of practice. Front. Physiol., 3, 326. 22934074 10.3389/fphys.2012.00326 PMC3429094

[btz190-B16] Spindel J.E., McCouch S.R. (2016) When more is better: how data sharing would accelerate genomic selection of crop plants. New Phytol., 212, 814–826. 27716975 10.1111/nph.14174

[btz190-B17] Wilkinson M.D. et al (2016) The FAIR guiding principles for scientific data management and stewardship. Sci. Data, 3, 160018. 26978244 10.1038/sdata.2016.18 PMC4792175

[btz190-B18] Wilkinson M.D., Links M. (2002) BioMOBY: an open source biological web services proposal. Brief. Bioinform., 3, 331–341. 12511062 10.1093/bib/3.4.331

[btz190-B19] Xin J. et al (2018) Cross-linking biothings APIs through JSON-LD to facilitate knowledge exploration. BMC Bioinformatics, 19, 30. 29390967 10.1186/s12859-018-2041-5 PMC5796402

